# 131 CYP24A1 Is Overexpressed in Keloid Keratinocytes and Its Inhibition Reduces Profibrotic Gene Expression

**DOI:** 10.1093/jbcr/irae036.130

**Published:** 2024-04-17

**Authors:** Dorothy M Supp, Jennifer M Hahn, Kelly A Combs, Caitlin Phillips, Heather M Powell

**Affiliations:** University of Cincinnati College of Medicine, Cincinnati, Ohio; Shriners Children's Ohio, Dayton, Ohio; The Ohio State University, Columbus, Ohio; University of Cincinnati College of Medicine, Cincinnati, Ohio; Shriners Children's Ohio, Dayton, Ohio; The Ohio State University, Columbus, Ohio; University of Cincinnati College of Medicine, Cincinnati, Ohio; Shriners Children's Ohio, Dayton, Ohio; The Ohio State University, Columbus, Ohio; University of Cincinnati College of Medicine, Cincinnati, Ohio; Shriners Children's Ohio, Dayton, Ohio; The Ohio State University, Columbus, Ohio; University of Cincinnati College of Medicine, Cincinnati, Ohio; Shriners Children's Ohio, Dayton, Ohio; The Ohio State University, Columbus, Ohio

## Abstract

**Introduction:**

Keloids are aggressive fibroproliferative lesions that can occur following skin injuries, including burns. Keloids negatively impact quality of life and are extremely challenging to treat. A better understanding of the underlying molecular mechanisms is needed for development of improved keloid therapies. We previously identified reduced vitamin D receptor (VDR) expression in keloid epidermis, implicating vitamin D and VDR-mediated signaling in keloid pathology. To investigate vitamin D signaling in keloid-derived cells, the current study analyzed expression of VDR and VDR target genes cathelicidin antimicrobial peptide (CAMP), cluster of differentiation 14 (CD14), endothelin 1 (EDN1), and 24 hydroxylase (encoded by CYP24A1) in normal and keloid keratinocytes. CAMP and CD14 are involved in innate immunity, EDN1 plays a role in endothelial dysfunction and fibrosis, and CYP24A1 is a vitamin D metabolizing enzyme that limits the amount of 1,25-dihydroxyvitamin D3 (1,25-D3), the active form of vitamin D. Additionally, the effects of 1,25-D3 treatment and CYP24A1 inhibition on expression of these genes, and other genes known to be aberrantly expressed in keloids, were investigated.

**Methods:**

Normal skin (N=6) and keloid (N=8) samples were obtained with IRB approval and primary keratinocyte cultures were established. For some experiments, keloid keratinocytes (N=3) were cultured +/- 1,25-D3, and +/- ketoconazole, which inhibits CYP24A1 enzyme activity. Gene expression was measured using quantitative PCR. Proliferation was measured via MTT assay. Statistical analyses were performed using t test (2 groups) or One Way ANOVA (>2 groups) using SigmaPlot 15.0.

**Results:**

Despite reduced VDR in keloid epidermis in vivo, keloid keratinocytes (KKs) in vitro expressed VDR mRNA at levels similar to normal keratinocytes (NKs) and responded to 1,25-D3 treatment with appropriate changes in target gene expression. Notably, in the absence of 1,25-D3 treatment, CYP24A1 expression in KKs was significantly higher than in NKs. Inhibition of CYP24A1 activity in KKs via ketoconazole treatment enhanced the effects of 1,25-D3 on expression of some target genes: levels of CYP24A1 and CD14 were increased, whereas EDN1 was significantly decreased. Ketoconazole treatment alone decreased expression of hyaluronan synthase 2 (HAS2) and periostin (POSTN), two profibrotic genes known to be overexpressed in keloid cells. Further, ketoconazole significantly reduced keratinocyte proliferation.

**Conclusions:**

The data suggest that CYP24A1 inhibition with ketoconazole may reduce 1,25-D3 inactivation, thereby enhancing the effects of 1,25-D3 on VDR target gene expression.

**Applicability of Research to Practice:**

Vitamin D has numerous anti-inflammatory, antiproliferative, and antifibrotic properties. Ketoconazole may potentiate the antifibrotic effects of 1,25-D3 and may thereby serve as an adjunctive therapy for suppression of keloid-associated gene expression changes.

